# Prehospital Buprenorphine: A National Cross-Sectional Analysis

**DOI:** 10.5811/westjem.46583

**Published:** 2026-10-11

**Authors:** Kira Chandran, Scott G. Weiner, Scott A. Goldberg, Vincent L. Holstein, Jane M. Hayes, Rebecca E. Cash

**Affiliations:** * Massachusetts General Hospital, Department of Emergency Medicine, Boston, Massachusetts; † Brigham and Women’s Hospital, Department of Emergency Medicine, Boston, Massachusetts; ‡ Harvard Medical School, Boston, Massachusetts; § Massachusetts General Hospital, Department of Psychiatry, Boston, Massachusetts; || McLean Hospital, Department of Psychiatry, Belmont, Massachusetts

## Abstract

**Introduction:**

Initiation of buprenorphine reduces mortality in people with opioid use disorder. However, patients who decline hospital transport after overdose may not be connected to treatment. To augment treatment engagement, prehospital buprenorphine pilot programs began in 2019. To date, few studies have looked at these programs nationally. Our objective was to evaluate national prehospital buprenorphine usage.

**Methods:**

We conducted a cross-sectional evaluation of emergency medical service (EMS) encounters involving prehospital buprenorphine administration using the 2020–2023 National EMS Information System Public Release Research datasets. Our primary outcome was trends in prehospital buprenorphine usage. Quarterly rates of prehospital buprenorphine were evaluated using joinpoint regression to identify inflection points. The secondary outcome was patient demographics and encounter characteristics, which were analyzed using descriptive statistics.

**Results:**

Of all EMS encounters with patient contact in the dataset (*N* = 167,221,959), there were 2,180 encounters where buprenorphine was administered (62% male [*n* = 1,349], mean age 37 years, standard deviation [SD] 10), with a significant increase per year (2020: *n* = 33; 2023: *n* = 1,574). Approximately half (52%; *n* = 1,125) of prehospital buprenorphine administrations occurred during 9-1-1 responses. Most encounters occurred in the South Atlantic region (4 administrations/100,000 encounters) and urban areas (72%; *n* = 1,572). Nearly all EMS crews were advanced life support (ALS) level of care (paramedic: 53% [*n* = 1,155]; ALS-community paramedic: 46% [*n* = 1,008]). Initial dosages of administered buprenorphine were 8 milligrams (mg) (45%; *n* = 973), 16 mg (36%; *n* = 786), or 24 mg (7%; *n* = 160). Most patients (80%; *n* = 1,746) had documented improvement with only four (0.2%) worse after administration. Few 9-1-1 encounters (16%; *n* = 180) resulted in hospital transport by EMS. Only 7% (*n* = 82) of 9-1-1 prehospital buprenorphine encounters had documented naloxone administration prior to buprenorphine dosing. There was a significant increase in number of buprenorphine administrations in quarter 2 of 2022 (slope increase of 54 administrations [95% CI, 20–89] per quarter versus 1 administration [95% CI, −1 to +3] per quarter from 2020–2022 quarter 1).

**Conclusion:**

Rates of prehospital buprenorphine have increased significantly between 2020 and 2023. Buprenorphine administration largely resulted in documentation of improvement in patient condition. With continued high rates of opioid-related deaths, prehospital buprenorphine may be considered by EMS agencies to prevent deaths from subsequent opioid use and connect patients to treatment.

## INTRODUCTION

Opioid-related overdose deaths in the United States peaked in 2023 with over 80,000 deaths.^1^ Emergency medical services (EMS) clinicians are often a primary touchpoint for people with opioid use disorder (OUD), such as in the case of an opioid overdose where they may be called to administer naloxone, an opioid reversal agent, and resuscitate the patient. One of 100 EMS calls within the US now involves the administration of naloxone.^2^ There have been increased rates of transport refusal by patients after nonfatal overdoses reversed with naloxone by EMS or a bystander, ranging from 22% to 73% declining transport in recent studies.^3^,^4^ Although the short-term mortality for those who decline hospital transport after overdose is relatively low, the risk of subsequent nonfatal overdose is high.^5^,^6^ Additionally, the 1-year mortality for patients with substance use disorder is exceedingly high. One study showed those who had received prehospital naloxone after overdose had a 1-year mortality of 7.5% (with exclusion of original overdose/immediate postoverdose period), highlighting the seriousness of substance use disorder as a disease itself.^7^

Promising ways to improve patient outcomes for people with OUD have largely involved medications for OUD, including buprenorphine and methadone. The initiation of buprenorphine by emergency physicians in people with OUD showed a 62% reduction in mortality at 1-year after overdose and a significant decrease in emergency department (ED) utilization rates for opioid-related visits at 30 days after overdose (58% repeat visit for opioid-related event compared to 38%).^8^,^9^ However, the opportunity to engage those with substance use disorder in treatment options extends far beyond the ED. Complex social factors (eg, housing instability, high financial cost, discontinuity of care) complicate the ability to adequately care for patients with substance use disorder from the ED alone.

Given the high rate of EMS transport refusal to a hospital after an overdose, initiation of buprenorphine by EMS clinicians in the field may be opportune.^9^ In 2019, New Jersey became the first state to include buprenorphine in their EMS protocols, leading to the first pilot program of prehospital buprenorphine.^10^ In this protocol, EMS clinicians could administer buprenorphine in the setting of precipitated withdrawal after an overdose reversal with naloxone on scene. 10 Since then, many EMS agencies and programs have followed suit with implementation of prehospital buprenorphine programs.^11^–^14^ This includes on-scene treatment for precipitated withdrawal and follow-up care; in some protocols EMS clinicians will return to the site of the nonfatal overdose 24–48 hours after the event to offer buprenorphine for patients who had initially declined treatment and hospital transport.^11^–^14^

A previous scoping review of prehospital buprenorphine programs conducted in 2024 found that 19 US states and Washington, DC had a program within their state, although their documentation was limited.^14^ If incorporated with a strong holistic approach to care including social work involvement and connection to outpatient clinicians, prehospital buprenorphine has been associated with significant improvement in patients staying in treatment for OUD and reduction in ED visits.^15^ However, little is known about the prevalence, usage, demographics, and trends in its use on the national level in the years following initiation. Our objective was to provide a cross-sectional analysis of national EMS encounters for prehospital buprenorphine between 2020 and 2023.

**Population Health Research Capsule**

What do we already know about this issue?

*Buprenorphine reduces mortality in opioid use disorder.*

What was the research question?

*What are national trends and characteristics of prehospital buprenorphine administration between 2020 and 2023?*

What was the major finding of the study?

*Prehospital buprenorphine administration increased between 2020*–*2023, rising in 2022 quarter 2 +54 per quarter (95% CI, 20*–*89) vs +1 per qtr pre-2022 Q2 (95% CI, −1 to +3).*

How does this improve population health?

*The expansion of prehospital buprenorphine may connect high-risk patients to treatment earlier and help prevent future opioid-related deaths.*

## METHODS

### Study Design and Setting

A retrospective, cross-sectional analysis was conducted using data from the 2020–2023 National EMS Information System (NEMSIS) Public Release Research datasets version 3.4.0. The NEMSIS is a deidentified, public release dataset including over 54 million EMS encounters annually as of 2023.^16^ Participation in the database varies by EMS agency and state, with estimates of 42–100% of agencies participating per state.^17^ This study was reviewed by a human subjects research review board and deemed exempt from review. Strengthening the Reporting of Observational Studies in Epidemiology (STROBE) guidelines were followed for this retrospective, cross-sectional analysis. This study was a secondary analysis of a clinical dataset derived from structured fields in the EMS electronic health record;^18^–^20^ no investigator chart review or manual data abstraction was performed.

### Selection of Emergency Medical Services Encounters

The study included all EMS encounters in the NEMSIS database between January 2020 and December 2023 where prehospital buprenorphine administration was documented. Encounters included 9-1-1 responses, as well as non–9-1-1 expanded scope events such as elderly assistance, injury prevention, public education, and immunization programs, often delivered by mobile integrated healthcare programs. The breakdown of non–9-1-1 expanded scope event subcategories is not available within this dataset.

### Measures

Our primary outcome was to evaluate trends in prehospital buprenorphine use over time and identify an inflection point in usage. We conducted a joinpoint regression of quarterly rates of use. Trends over time were evaluated and inflection points identified. Our secondary outcome was to evaluate patient demographics and encounter characteristics of prehospital buprenorphine usage. Patient demographics including sex, race, ethnicity, age, US Census division, and urbanicity were included. We evaluated the following encounter characteristics: level of care; type of service requested; EMS scene time; role person administering medication; primary impression; primary symptoms; and patient disposition. Additionally, buprenorphine dosages, number of doses, and response to medication were evaluated.

Consistent with previous studies, we identified rates of patients who received both naloxone and buprenorphine in the same encounter to assess the use of buprenorphine in precipitated withdrawal. 10 All naloxone doses administered during the encounter were identified and compared with the time of buprenorphine administration to determine whether naloxone was provided before or after the initial dose of buprenorphine.

### Data Analysis

We calculated quarterly rates of buprenorphine administration per year. We then performed joinpoint regression analysis to identify inflection points in prehospital buprenorphine administration. A segmented regression was fitted. Confidence intervals were calculated at 95%. We created a graph using RStudio. software (Posit PBC, Boston, MA). Patient demographics and EMS encounter characteristics for encounters involving buprenorphine administration were evaluated using descriptive statistics. We then performed a subdivision analysis between 9-1-1 response encounters and non–9-1-1 response encounters.

We calculated rates of prehospital buprenorphine administration per 100,000 EMS encounters with patient contact within each US Census division over the time of the study. A map was created using RStudio showing rates of prehospital buprenorphine administration per census division. We produced the map using Albers equal-area conic projection. State level data were not available in NEMSIS for review.

## RESULTS

### Trends in National Prehospital Buprenorphine Use

There were 167,221,959 total EMS encounters with patient contact in the dataset. Of those, 2,180 included EMS encounters with documented buprenorphine administration. There was a significant increase in the number of buprenorphine administrations starting in quarter 2 (Q2) of 2022 (Figure 1). The slope increased by 54 buprenorphine administrations per quarter [95% CI, 20–89] after Q2 of 2022 through 2023. Prior to Q2 of 2022, the slope increased by one administration of buprenorphine per quarter [95% CI, **−**1 to +3] from 2020 to Q1 of 2022.

### Patient Demographic and Regional Distribution of Buprenorphine Use

There were 1,349 EMS encounters for male patients (62%) and 814 EMS encounters for female patients (37%) who received prehospital buprenorphine (Table 1). The mean age of patients was 37 years (standard deviation [SD] 9.8). Most buprenorphine administrations happened in urban areas (1,572 encounters, 72%), with lower rates in suburban (590 encounters, 27%), and rural areas (1 encounter, 0%). Patient race was documented for < 1% of the encounters and was therefore excluded from analysis.

There were four prehospital buprenorphine administrations per 100,000 EMS encounters, with patient contact in the South Atlantic region far exceeding reported use in all other US Census divisions (< 1 prehospital buprenorphine administration/100,000 EMS encounters) (Figure 2). Approximately half (1,042, 48%) of all prehospital buprenorphine encounters within the dataset were noted to be public assistance encounters, which comprised most (> 99%) of the non–9-1-1 subdivision and were found to be exclusively from the South Atlantic region.

### Emergency Medical Services Encounter Characteristics for Prehospital Buprenorphine Administration

As noted above, EMS encounters that included prehospital buprenorphine administration were split between 9-1-1 responses (1,125 encounters, 52%) and non–9-1-1 responses (1,055 encounters, 48%; Table 2). Within the non-9-1-1 subdivision, 1,042 of 1,055 encounters (> 99%) were for public assistance/other not listed. There were only nine encounters (< 1% of the non–9-1-1 subdivision) that were for other types of transfer including interfacility transports, mutual aid, medical transports, or intercepts. Most encounters were with advanced life support (ALS) personnel. A total of 1,155 encounters included paramedics (53%), and 1,008 encounters included community paramedicine paramedics (46%). Only 17 calls (0.8%) were listed as basic life support (BLS) personnel. The mean EMS scene time was 40.5 minutes (SD 25.5). A minority of the encounters resulted in transport to the hospital. Many encounters, (960, 44%) were “Patient Treated, Released (per protocol)” with 174 (8%) “Patient Evaluated, No Treatment/Transport Required.” A total of 786 encounters (36%) were “Assist, Public” or “Assist, Unit.” During only 185 encounters (8%) patients were treated and transported by the EMS unit, with most transfers occurring after 9-1-1 calls.

The most common primary impression was “Mental and behavioral disorders due to multiple drug use and use of other psychoactive substance” seen in 434 encounters (20%) and “Mental and behavioral disorders due to use of opioids” for 433 encounters (20%). Primary impression was not reported for 39% of encounters.

### Buprenorphine Dosage, Response, and Naloxone Rates

The most common initial dose of buprenorphine was 8 mg (973 doses, 45%), followed by 16 mg (786 doses, 36%), and 24 mg (160 doses, 7%; Table 2). Most encounters had documentation of improvement after administration of first dose of buprenorphine (1,746 encounters, 80%), and only 4 patients (0.2%) were documented as worse after the first dose of buprenorphine. There were 222 encounters (10%) where more than one dose of buprenorphine was administered during the same encounter. Of the encounters where a second dose of buprenorphine was administered, 143 encounters (64%) documented improvement after the second dose administration, 36 (16%) documented unchanged response, 42 (19%) lacking documented response, and one encounter (< 1%) with documented worse response after second dose.

In only 85 encounters (4% of all EMS encounters, and 7% of 9-1-1 encounters), both naloxone and buprenorphine were administered during the same encounter (including documented bystander naloxone administration); 34 of those (42%) had received at least two doses of naloxone. In all these encounters, patients received naloxone prior to administration of buprenorphine according to the timing of medication administration, suggesting that buprenorphine had been given in the setting of naloxone-precipitated opioid withdrawal.

## DISCUSSION

Given the mortality benefit shown in ED initiation of buprenorphine to treat people with OUD, the expansion of buprenorphine administration to the prehospital setting is a promising strategy to further reduce opioid-related deaths. The implementation of prehospital buprenorphine has increased nationally since its origins in 2019.^8^ We found an inflection point during 2022, with a rapid increase in quarterly buprenorphine administration. This is likely attributed to an increasing number of agencies implementing these programs in 9-1-1 systems and via community paramedicine.^11^,^13^ The patient demographics of those receiving buprenorphine is predominately male, median age range in the mid-30s, and mirrors the demographic of patients being treated for OUD in EDs nationally, showing an opportunity to engage this patient population in a similar way via prehospital intervention.^21^

This study provides insight into situations when prehospital buprenorphine is being administered, which there is scarce documentation of nationally. Indications for prehospital buprenorphine use have typically included administration after precipitated withdrawal from naloxone, which is used to reverse opioid overdose.^10^ By alleviating the symptoms of naloxone-precipitated withdrawal through buprenorphine administration, it is hypothesized that patients may be more likely to initiate treatment, continue treatment for dependence, and engage with recovery programs.^22^ Our work shows only a minority of 9-1-1 encounters (7%) are for patients given buprenorphine after EMS or bystander naloxone administration. There may be a limitation of underreporting of naloxone administration. This highlights a large area of improvement and opportunity for prehospital buprenorphine initiation. There were an estimated 1,000 naloxone administrations per 100,000 EMS events in 2016 for opioid overdose, far exceeding the rates of prehospital buprenorphine use from even the highest usage geographic areas in this study.^2^

This study also provides insight into the prevalence of prehospital buprenorphine programs nationally. A recent review from 2024 documented 19 US states with programs and estimated that approximately 62% of the buprenorphine delivered was via 9-1-1, with 46% delivered by community paramedics 24–48 hours after overdose.^14^ Given the number of encounters involving public assistance in documented in that review, there is likely a large percentage of prehospital buprenorphine administration occurring via community paramedicine outreach programs, although that is difficult to quantify given lack of clarity within the dataset. Community paramedicine outreach programs often include both methods of buprenorphine administration for patients in naloxone-precipitated withdrawal, and secondary pathways for patients with recent opioid use.^13^ Follow-up for patients who decline treatment or transport is variable depending on the community setting and substance use- recovery infrastructure already in place. One study based in New Jersey looking at buprenorphine-equipped ambulances had a protocol to arrange next-day substance use follow-up for patients after suspected overdose events.^23^ A different EMS protocol in North Carolina included referrals to opioid treatment centers after prehospital buprenorphine administration. They also had an ability to continue dosing patients in the field by community paramedics for up to five days while awaiting a scheduled appointment.^13^ These teams can include community paramedics, peer recovery specialists, and social workers.^11^,^13^,^23^

We found that a minority of patients were transported to the hospital after treatment (only 16% of 9-1-1 encounters in this study). Without the touchpoint of the ED, EMS clinicians can play an important role in providing patients with resources and treatment for withdrawal on scene, which may increase the likelihood of starting and continuing in treatment. Prior work on community paramedicine initiation of medications for OUD showed that 64% of referred patients were still engaged in treatment at 30 days.^13^ Additionally, the ability to treat patients on scene for withdrawal may decrease ED utilization to provide immediate treatment for symptoms and reduce a barrier to treatment.

However, barriers to prehospital buprenorphine implementation include patient refusal of medication, difficulty in locating patients for follow-up if patient is unhoused, insurance and telehealth barriers, and operational barriers.^14^ Additionally, longer than average on-scene times were noted (40 minutes in this study, compared to cited averages ranging from 14–17 minutes in prior EMS literature).^24^,^25^ This has implications on EMS operations and 9-1-1 staffing models.

Most encounters had documentation of improved symptoms after buprenorphine administration, suggesting this is an effective and safe method for treating opioid withdrawal. Importantly, less than 1% of encounters had worse outcomes after administration of buprenorphine, indicated a low prevalence of buprenorphine-precipitated withdrawal. There is no standardization of starting dosages for prehospital buprenorphine, with EMS agencies largely adopting their own protocols.^14^ Nationally, there has been a recent push towards higher starting doses of buprenorphine, given the increasing prevalence of fentanyl in the nonmedical drug supply, which is associated with lower rates of future ED utilization.^26^ In this study, we found most starting dosages to be 8 mg and 16 mg, which is in line with current recommendations. It is notable that 10% of patients received a second dose of buprenorphine after the initial dose. The response to a second dose of buprenorphine also showed continued high rates of documented improvement in condition (64%) with less than 1% documented as worse after administration, suggesting utility in sequential dosing. However, the optimal starting dosage and protocol for prehospital buprenorphine is still unknown and will likely vary based on indication (eg precipitated withdrawal).

## LIMITATIONS

There are several limitations to this study. Although the NEMSIS database includes most EMS activations nationally, with over 50 million activations per year, not all EMS agencies participate in data collection. Further, nonemergency encounters such as an mobile integrated health or community paramedic visits may not be submitted to NEMSIS. These prehospital programs may document the details of the encounter outside of the EMS record, such as in their local hospital system electronic health record, limiting our ability to account for those administrations. Thus, it is likely that a portion of prehospital buprenorphine administrations were not included.

Additionally, the study is limited to the characteristics provided in the NEMSIS database, therefore outcome data and nuanced analysis of the situations and encounters is not available for analysis. Although we analyzed the EMS clinician’s primary impression, the broad “primary impression” terms and missing primary impression data (39%) make it difficult to interpret the reason for EMS activation and buprenorphine administration per encounter. And because the number of encounters for opioid overdose was not evaluated in this analysis, the proportion of buprenorphine administrations per overdose could not be assessed.

Additionally, the inclusion of all EMS encounters and not 9-1-1 only encounters likely led to an over-representation of the South Atlantic region in this dataset, given that all of the public assistance encounters, which was the majority of the non-9-1-1 encounters, came from that area. This may be secondary to geographic differences in documentation and coding within NEMSIS versus implementation and integration of mobile integrated health networks into NEMSIS records. It should be noted the public assistance encounters (majority of non–9-1-1 encounters) in the dataset includes a substantial portion of suburban data, with 9-1-1 only encounters being almost exclusively urban, and provides additional insight into prehospital buprenorphine use, which is why we have kept this within the dataset.

## CONCLUSION

This retrospective study showed increased rates of prehospital buprenorphine usage across the US from 2020 to 2023, with a sharp increase in 2022. About half of encounters were for patients treated after 9-1-1 response, but few received naloxone prior to buprenorphine, suggesting an expansion of prehospital buprenorphine use from the original conception of use after precipitated withdrawal. Prehospital buprenorphine was documented to improve the patient’s condition for most encounters, with very few documented as having a poor response. The expanding scope of practice allowing EMS clinicians to engage in harm reduction strategies with prehospital buprenorphine may be an important opportunity to prevent opioid overdose deaths and connect patients to treatment. Further studies are needed to evaluate long-term patient outcomes.

## Figures and Tables

**Figure 1:**
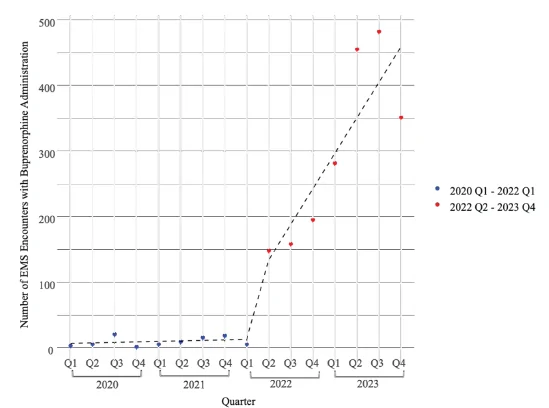
Joinpoint regression of national prehospital buprenorphine quarterly administrations from 2020–2023 is shown in a retrospective study of emergency medical services encounters using the National EMS Information System database. An inflection point is present in Quarter 2 2022. *EMS*, emergency medical services; *Q*, quarter.

**Figure 2:**
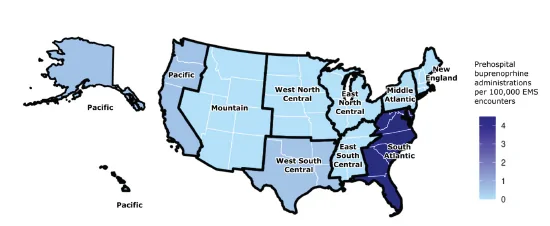
Rates of prehospital buprenorphine administration per 100,000 EMS emergency medical services encounters with patient contact between 2020 and 2023 for each US Census division in a retrospective study using the National EMS Information System database. Map produced using Albers equal-area conic projection. *EMS*, emergency medical services.

**Table 1:** Patient demographics and encounter geographics of emergency medical services encounters involving prehospital buprenorphine administration nationally between 2020 and 2023 in a retrospective study using the National EMS Information System database.

Demographic	All	9-1-1	Non–9-1-1
Patient sex, *n* (%)			
Male	1,349 (62%)	724 (64%)	625 (59%)
Female	814 (37%)	386 (34%)	428 (41%)
Patient age in years, mean (SD)	37 (9.8)	38 (10.8)	37 (8.6)
US Census Division, *n* (%)			
South Atlantic	1,922 (88%)	872 (78%)	1,050 (100%)
Pacific	121 (6%)	121 (11%)	0
West South Central	104 (5%)	101 (9%)	3 (< 1%)
Middle Atlantic	27 (1%)	27 (2%)	0
Other (East North Central, West North Central, New England, West South Central, Mountain Divisions)	6 (< 1%)	4 (< 1%)	2 (< 1%)
Urbanicity, *n* (%)			
Urban	1,572 (72%)	1,102 (98%)	470 (45%)
Suburban	590 (27%)	9 (1%)	581 (55%)
Rural	1 (< 1%)	0	1 (< 1%)

*SD*, standard deviation.

**Table 2:** Emergency medical services encounter characteristics involving prehospital buprenorphine administration nationally between 2020 and 2023 in a retrospective study using the National EMS Information System database.

EMS Encounter Characteristics	All	9-1-1	Non–9-1-1
Level of care, *n* (%)			
Paramedic	1,155 (53%)	627 (56%)	528 (50%)
Community paramedicine paramedic	1,008 (46%)	486 (43%)	522 (50%)
EMT	12 (< 1%)	10 (1%)	2 (< 1%)
Community paramedicine EMT	5 (< 1%)	2 (< 1%)	3 (< 1%)
Type of service requested, *n* (%)			
9-1-1 Response	1,125 (52%)	1,125 (100%)	0
Public assistance/other not listed	1,042 (48%)	0	1,042 (99%)
Interfacility transport	9 (< 1%)	0	9 (1%)
Mutual aid, intercept, medical transport	4 (< 1%)	0	4 (< 1%)
EMS scene time, *n* (%)			
Mean (SD)	40.5 min (25.5)	36.5 min (8.5)	42.7 min (25.1)
Patient disposition, *n* (%)			
Patient treated, released (per protocol)	960 (44%)	180 (16%)	780 (74%)
Public assistance	770 (35%)	640 (57%)	130 (12%)
Treated and transported by EMS	185 (8%)	180 (16%)	5 (< 1%)
Patient evaluated, no treatment/transport required	174 (8%)	54 (5%)	120 (11%)
Patient refused care and/or transport	52 (2%)	44 (4%)	8 (1%)
Other	39 (2%)	27 (2%)	12 (1%)
1^st^ Buprenorphine dosage given, *n* (%)			
8 mg	973 (45%)	454 (40%)	519 (49%)
16 mg	786 (36%)	508 (45%)	278 (26%)
24 g	160 (7%)	122 (11%)	38 (4%)
12 mg	123 (6%)	11 (1%)	112 (11%)
4 mg	72 (3%)	7 (1%)	65 (6%)
2 mg	18 (1%)	5 (< 1%)	13 (1%)
20 mg	18 (1%)	0	18 (2%)
Other	30 (1%)	18 (2%)	12 (2%)
Response to medication (buprenorphine), *n* (%)			
Improved	1,746 (80%)	832 (74%)	914 (87%)
Unchanged	358 (16%)	233 (21%)	125 (12%)
Worse	4 (< 1%)	2 (< 1%)	2 (< 1%)
Not recorded/not applicable	72 (3%)	58 (5%)	14 (1%)
Naloxone given prior to buprenorphine, *n* (%)	85 (4%)	82 (7%)	3 (< 1%)

*EMS*, emergency medical services*; EMT*, emergency medical technician; *SD*, standard deviation.

## References

[1] National Institute on Drug Abuse Drug Overdose Deaths: Facts and Figures.

[2] Cash RE, Kinsman J, Crowe RP (2019). Naloxone administration frequency during emergency medical service events—United States, 2012–2016. Morb Mortal Wkly Rep.

[3] Rock P, Singleton M (2019). EMS heroin overdoses with refusal to transport & impacts on ED overdose surveillance. Online J Public Health Inform.

[4] Jacoves J, Mannerings A, Stader D (2026). Assessment of a county-wide, pan-first responder take-home naloxone program in Denver, Colorado. JACEP Open.

[5] Levine M, Sanko S, Eckstein M (2016). Assessing the risk of prehospital administration of naloxone with subsequent refusal of care. Prehosp Emerg Care.

[6] Zozula A, Neth MR, Hogan AN (2022). Non-transport after prehospital naloxone administration is associated with higher risk of subsequent non-fatal overdose. Prehosp Emerg Care.

[7] Weiner SG, Baker O, Bernson D (2022). One-year mortality of patients treated with naloxone for opioid overdose by emergency medical services. Subst Abus.

[8] Samples H, Nowels MA, Williams AR (2023). Buprenorphine after nonfatal opioid overdose: reduced mortality risk in Medicare disability beneficiaries. Am J Prev Med.

[9] Skains RM, Reynolds L, Carlisle N (2023). Impact of emergency department–initiated buprenorphine on repeat emergency department utilization. West J Emerg Med.

[10] Carroll GG, Wasserman DD, Shah AA (2021). Buprenorphine field initiation of ReScue treatment by emergency medical services (Bupe FIRST EMS): A case series. Prehosp Emerg Care.

[11] Hern HG, Lara V, Goldstein D (2023). Prehospital buprenorphine treatment for opioid use disorder by paramedics: First year results of the EMS buprenorphine use pilot. Prehosp Emerg Care.

[12] Champagne-Langabeer T, Bakos-Block C, Yatsco A (2020). Emergency medical services targeting opioid use disorder: an exploration of current out-of-hospital post-overdose interventions. JACEP Open.

[13] Belden C, Kopak A, Coules C (2024). Building bridges to outpatient treatment services for post-overdose care via paramedic buprenorphine field initiation. J Subst Use Addict Treat.

[14] Gormley MA, Moschella P, Pham T (2025). Implementation barriers of prehospital buprenorphine administration programs in the United States: a scoping review. Prehosp Emerg Care.

[15] D’Onofrio G, O’Connor PG, Pantalon MV (2015). Emergency department–initiated buprenorphine/naloxone treatment for opioid dependence: a randomized clinical trial. JAMA.

[16] National EMS Information System (NEMSIS) Request research data.

[17] Duong HV, Herrera LN, Moore JX (2018). National characteristics of emergency medical services responses for older adults in the United States. Prehosp Emerg Care.

[18] National Highway Traffic Safety Administration, Office of EMS National EMS Information System (NEMSIS).

[19] Ehlers J, Fisher B, Peterson S (2022). Description of the 2020 NEMSIS public-release research dataset. Prehosp Emerg Care.

[20] National Emergency Medical Services Information System (NEMSIS) NEMSIS version 3 extended data definitions.

[21] Herring AA, Rosen AD, Samuels EA (2024). Emergency department access to buprenorphine for opioid use disorder. JAMA Netw Open.

[22] Herring AA (2020). Postoverdose initiation of buprenorphine after naloxone-precipitated withdrawal is encouraged as a standard practice in the California Bridge Network of Hospitals. Ann Emerg Med.

[23] Carroll G, Solomon KT, Heil J (2023). Impact of administering buprenorphine to overdose survivors using emergency medical services. Ann Emerg Med.

[24] Texas Department of State Health Services Texas health data—emergency medical service (EMS) 911 run times.

[25] Lawner BJ, Szabo K, Daly J (2020). Prehospital trauma scene and transport times for pediatric and adult patients. West J Emerg Med.

[26] Axeen S, Pacula RL, Merlin JS (2024). Association of daily doses of buprenorphine with urgent health care utilization. JAMA Netw Open.

